# Sensitivity evaluation of 2019 novel coronavirus (SARS-CoV-2) RT-PCR detection kits and strategy to reduce false negative

**DOI:** 10.1371/journal.pone.0241469

**Published:** 2020-11-18

**Authors:** Yunying Zhou, Fengyan Pei, Mingyu Ji, Li Wang, Huailong Zhao, Huanjie Li, Weihua Yang, Qingxi Wang, Qianqian Zhao, Yunshan Wang

**Affiliations:** 1 Medical Research & Laboratory Diagnostic Center, Jinan Central Hospital, Cheeloo College of Medicine, Shandong University, Jinan, Shandong, China; 2 Microbiology Department, Jinan Central Hospital Affiliated to Shandong First Medical University, Jinan, Shandong, China; 3 Research Center of Basic Medicine, Jinan Central Hospital Affiliated to Shandong First Medical University, Jinan, Shandong, China; 4 Jinan Infectious Disease Hospital, Shandong University, Jinan, Shandong, China; 5 Jinan Center for Disease Control and Prevention, Jinan, Shandong, China; Faculty of Science, Ain Shams University (ASU), EGYPT

## Abstract

The early detection and differential diagnosis of respiratory infections increase the chances for successful control of COVID-19 disease. The nucleic acid RT-PCR test is regarded as the current standard for molecular diagnosis. However, the maximal specificity confirmation target *ORF1ab* gene is considered to be less sensitive than other targets in clinical application. In addition, recent evidence indicated that the initial missed diagnosis of asymptomatic patients with SARS-CoV-2 and discharged patients with “re-examination positive” might be due to low viral load, and the ability of rapid mutation of SARS-CoV-2 also increases the rate of false-negative results. Moreover, the mixed sample nucleic acid detection is helpful in seeking out the early community transmission of SARS-CoV-2 rapidly, but the detection kit needs ultra-high detection sensitivity. Herein, the lowest detection concentration of different nucleic acid detection kits was evaluated and compared to provide direct evidence for the selection of kits for mixed sample detection or make recommendations for the selection of validation kit, which is of great significance for the prevention and control of the current epidemic and the discharge criteria of low viral load patients.

## Introduction

The coronavirus that caused the outbreak was identified in the case of viral pneumonia in Wuhan in 2019 [1–3] and was named as 2019-nCoV/SARS-CoV-2 by the World Health Organization (WHO) [2–5]. SARS-CoV-2 belongs to the coronavirus genus β with a single-stranded, non-segmented positive-sense RNA genome [6], which is the seventh known coronavirus that can infect humans [1, 7]. Similar to the other pathogenic RNA viruses, the genetic RNA material is the earliest marker to be detected. The nucleic acid detection or sequencing is currently used in conjunction with pulmonary CT for the clinical diagnosis of COVID-19 [8, 9]. As the disease progresses, antibodies IgM and IgG were produced by the human immune system. Although antibody tests play a major role in monitoring the response to future immunization strategies and demonstrating previous exposure/immunity, the antibody-positive rate often lags behind the nucleic acid detection [10–12], and the cross-reactions were detected in SARS-CoV antigen with autoantibodies [13].

Theoretically, real-time PCR detection is widely used as the molecular diagnosis standard for SARS-CoV-2 [14, 15]. Recently, the analysis showed that the pattern of viral load change in COVID-19 patients was similar to that in patients with influenza; however, it was different from that in SARS and MERS (whose viral load peaked about 10 days after the onset of symptoms) [16–19]. In COVID-19 patients, RT-PCR was detected as positive one day before the onset of symptoms, while most COVID-19 patients cannot be detected before premorbid because of the low copy number of the virus [7, 17, 20]. In addition, some discharged patients appearing “re-examination-positive” could be attributed to the persistence of a small number and low copy number of the viruses. However, the false-negative rate is about 20–40% in the highly suspicious cases in China [21–23] due to improper sample collection, storage, personnel operation, and low sensitivity test kit [24]. Furthermore, mutation or deletion in the target gene leads to false-negative results [25, 26].

The current RT-PCR nucleic acid detection has not only a high false-negative rate but also a low sensitivity rate [23]. The target genes for SARS-CoV-2 are based on the conserved and specific open reading frame 1ab (*ORF1ab*), spike (*S*), RNA-dependent RNA polymerase (*RdRp*), envelope (*E*), and nucleocapsid (*N*) genes [6, 27–31]. Although *ORF1ab* is the highest specificity confirmation target gene, it is considered to be less sensitive than other targets in clinical application [28]. Recently, mixed sample testing has been widely used in large-scale population testing (http://www.nhc.gov.cn/yzygj/s7659/202008/fa5057afe4314ef8a9172edd6c65380e.shtml). However, some studies propose that although this method improves the detection efficiency, it might miss the individuals with low viral load [29, 32]. In addition, none of the approved nucleic acid test kits mentioned whether they could be used for mixed sample detection. Thus, the pattern of ORF1ab-positive reports cause missed tests? Is it feasible to report based on positive *N* or *E* genes? Clinically, it is recommended that samples with suspicious results or single-channel positive results should be re-examined with another manufacturer’s kit or method. Nonetheless, the basis for selecting a specific validation kit is yet to be elucidated.

## Materials and methods

### Patients

A total of 10 confirmed cases of COVID-2019 patients (2 females and 8 males, 5–50-years-old) were collected from January to February 2020, in Jinan Central Hospital Affiliated to Shandong University and Jinan Infectious Disease Hospital, Shandong University. These patients were diagnosed based on the clinical symptoms, lung computed tomography (CT), and nucleic acid test. Also, 100 suspected cases (symptoms of fever, dry cough, and pneumonia images) were collected from the first institute listed above.

### Ethics statement

This study was approved by the Ethics Commission of Jinan Central Hospital. The written informed consent was obtained from all study participants, and the written parental consent were obtained from the minors before the study was begun.

### Specimen collection

The specimens were collected according to the guidelines of the Chinese Center for Disease Control and Prevention (CCDC) (http://www.chinacdc.cn/jkzt/crb/zl/szkb_11803/jszl_11815/202003/t20200309_214241.html). Nasopharyngeal and oropharyngeal swabs were inserted into one sterile tube containing 3 mL of virus preservation solution. The positive specimens were obtained from the patient 2 days after they were diagnosed as COVID-19. In the case of highly suspicious patients, the nucleic acid testing for SARS-CoV-2 was repeated on new nasopharyngeal samples and oropharyngeal swabs, and the serum for antibody testing was collected at an interval of 24 h, until it was confirmed to be positive or negative. In addition, environmental specimens were collected from the surface in direct contact with the patient, such as the inner side of the mask, phone, doorknob, and bedside. Each surface was wiped with one synthetic fiber swab that was then inserted into a sterile tube.

### Virus RNA extraction

Then RNA extraction, gradient dilution and PCR amplification were performed within two hours after specimen collection. The virus RNA was extracted using the magnetic beads method, according to the instructions of the nucleic acid extraction kit (Shanghai Zhijiang Biotechnology Co., Ltd, Shanghai, China), and then diluted with RNA extraction of negative samples. RNA concentration of the extraction were 20~100ng/μL detected by Nanodrop 2000.

### Real-time PCR

To verify the sensitivity of SARS-CoV-2 nucleic acid detection kits, five kits were selected. Kit-1 and kit-2 were designed according to the primer and probe sequences published by China’s CDC and were the first kits approved by the National Medical Products Administration (NMPA) and subsequently received European Confirmity (CE) marking. Kit-3 designed according to the primer and probe sequences published by WHO [6], was also approved by NMPA. Kit-4 and kit-5 were self-designed kits based on the genomes of SARS-CoV-2. The name, source, and catalog number of the five test kits are as follows: Kit-1 (BioGerm, Shanghai BioGerm Medical Co., Ltd, SJ-HX-226-1,2), Kit-2 (Liferiver, Shanghai Liferiver Biotech Co., Ltd, Z-RR-0479-02-50), Kit-3 (ACV, Shandong ACV Biotech Co., Ltd, B7200118-0102), Kit-4 (XABTBeijing ZCHS Biotech Co., Ltd, ZCHS-C-YF-JC19-01), and Kit-5 (bioPerfectus, Jiangsu bioPerfectus Biotech Co., Ltd, JC10223-1N). Each kit contained 25 μL of the reaction system, including 5 μL of the RNA template. The amplification reaction was set according to the instructions of the kits and carried out on the ABI7500 Real-time PCR system (Applied Biosystems, USA). The information for the five amplification kits is shown in Table 1.

**Table 1:** Information for the amplification kits of SARS-CoV-2.

Kit Name	primer and probes source	Amplification targets and region (amino acid)	Primers and probes Sequence		Ct value of suspicious region		Missense mutation
**Kit 1**	CDC	ORF1ab (4447–4487)		F: CCCTGTGGGTTTTACATTAA	35 to 38	**50**^**。**^**C 10min**	**ORF1ab:**
		N gene (9627–9660)	ORF1ab	P: FAM-CCGTCTGCGGTATGTGGAAAGGTTATGG-BHQ1		**↓**	A (117) → T
				R: ACGATTGTGCATCAGCTGA		**95**^**。**^**C 5min**	P (309) → S
						**↓**	S (428) → N
				F: GGGGAACTTCTCCTGCTAGAAT		**95**^**。**^**C 10s**	T (609) → I
			N gene	P: FAM-TTGCTGCTGCTTGACAGATT-TAMRA		**↓40cycles**	A (1176) → V
				R: CAGACATTTTGCTCTCAAGCTG		**55**^**。**^**C 40s**	L (1599) → F
**Kit 2**	WHO	RdRP (5143–5173)		F: GTGARATGGTCATGTGTGGCGG	40 to 43	**45**^**。**^**C 10min**	I (1607) → V
		N gene (8756–8794)		P2: FAM-CAGGTGGAACCTCATCAGGAGATGC-BBQ		**↓**	M (2194) → T
		N gene (9569–9611)	RdRP	P1: FAM-CCAGGTGGWACRTCATCMGGTGATGC-BBQ		**95**^**。**^**C 3min**	L (2235) → I
				R: CARATGTTAAASACACTATTAGCATA		**↓**	I (2244) → T
						**95**^**。**^**C 15s**	G (2251) → S
						**↓45cycles**	A (2345) → V
				F: ACAGGTACGTTAATAGTTAATAGCGT		**58**^**。**^**C 30s**	G (2534) → V
**Kit 3**	WHO	ORF1ab (5143–5173)	N gene	P1: FAM-ACACTAGCCATCCTTACTGCGCTTCG-BBQ	38 to 40	**45**^**。**^**C 10min**	D (2579) → A
		N gene (8756–8794)		R: ATATTGCAGCAGTACGCACACA		**↓**	N (2708) → S
		E gene (9569–9611)				**95**^**。**^**C 3min**	F (2908) → I
						**↓**	T (3058) → I
				F: CACATTGGCACCCGCAATC		**95**^**。**^**C 15s**	S (3099) → L
			E gene	P: FAM-ACTTCCTCAAGGAACAACATTGCCA-BBQ		**↓45cycles**	L (3606) → F
				R: GAGGAACGAGAAGAGGCTTG		**60**^**。**^**C 45s**	E (3764) → D
**Kit 4**	Self-Designed	ORF1ab		unknown	35 to 38	**45**^**。**^**C 20min**	N (3833) → K
		N gene				**↓**	W (5308) → C
		E gene				**95**^**。**^**C 10min**	T (5579) → I
						**↓**	I (6075) → T
						**95**^**。**^**C 15s**	P (6083) → L
						**↓40cycles**	F (6309) → Y
						**55**^**。**^**C 40s**	E (6565) → D
**Kit 5**	Self-Designed	ORF1ab		unknown	37 to 40	**50**^**。**^**C 10min**	K (6958) → R
		N gene				**↓**	D (7018) → N
		E gene				**97**^**。**^**C 1min**	**N gene:**
						**↓**	T (148) → I
						**97**^**。**^**C 5s**	S (194) → L
						**↓45cycles**	S (202) → N
						**58**^**。**^**C 30s**	P(34) → S

### Defined criteria of PCR results

The sensitivity comparison of multiple samples and kits should ensure that the initial concentration of samples is consistent. Then, to set the threshold and analyze each target separately for PCR amplification, the threshold should be adjusted to fall within the PCR exponential phase and higher than any background noise. Finally, the data were judged according to the Ct value and the shape of the curve, and the lowest detection concentration of positive data was used as the standard to judge the sensitivity of the kits. The positive result must be a typical S curve, and the Ct value must be within the positive reporting region according to each kit’s instructions (Table 1). For instance, the Ct value is ≤35 for kit-1 and kit-4, ≤40 for kit-2, ≤38 for kit-3, and ≤37 for kit-5.

### Continuous amplification

The RT-PCR products were re-amplified for another 40 cycles under the same amplification conditions. A total of 53 nucleic acid samples of other respiratory pathogens with known concentrations were used for specificity test.

### Laboratory quality-control

Respiratory specimens include nasopharyngeal and oropharyngeal swabs, bronchoalveolar lavage fluid, tracheal aspirates, and sputum. However, cotton swab heads are not allowed for swab specimens. Also, serum specimens were collected. Specimens should not be stored for more than 72 h at 4 °C. Positive and negative controls should be tested simultaneously with the samples. The fluorescence amplification curve of the negative control should not exceed the threshold. The Ct value of all the targets in the positive control should be within the expected range. The detection kit should contain the internal target gene, and the amplification curve should exceed the threshold line.

### Statistical methods

SPSS18.0 software was used for statistical analysis. The Student’s t-test was used to evaluate the differences between Ct values.

## Results

### Sensitivity evaluation of SARS-CoV-2 detection kits

**Fig 1:**
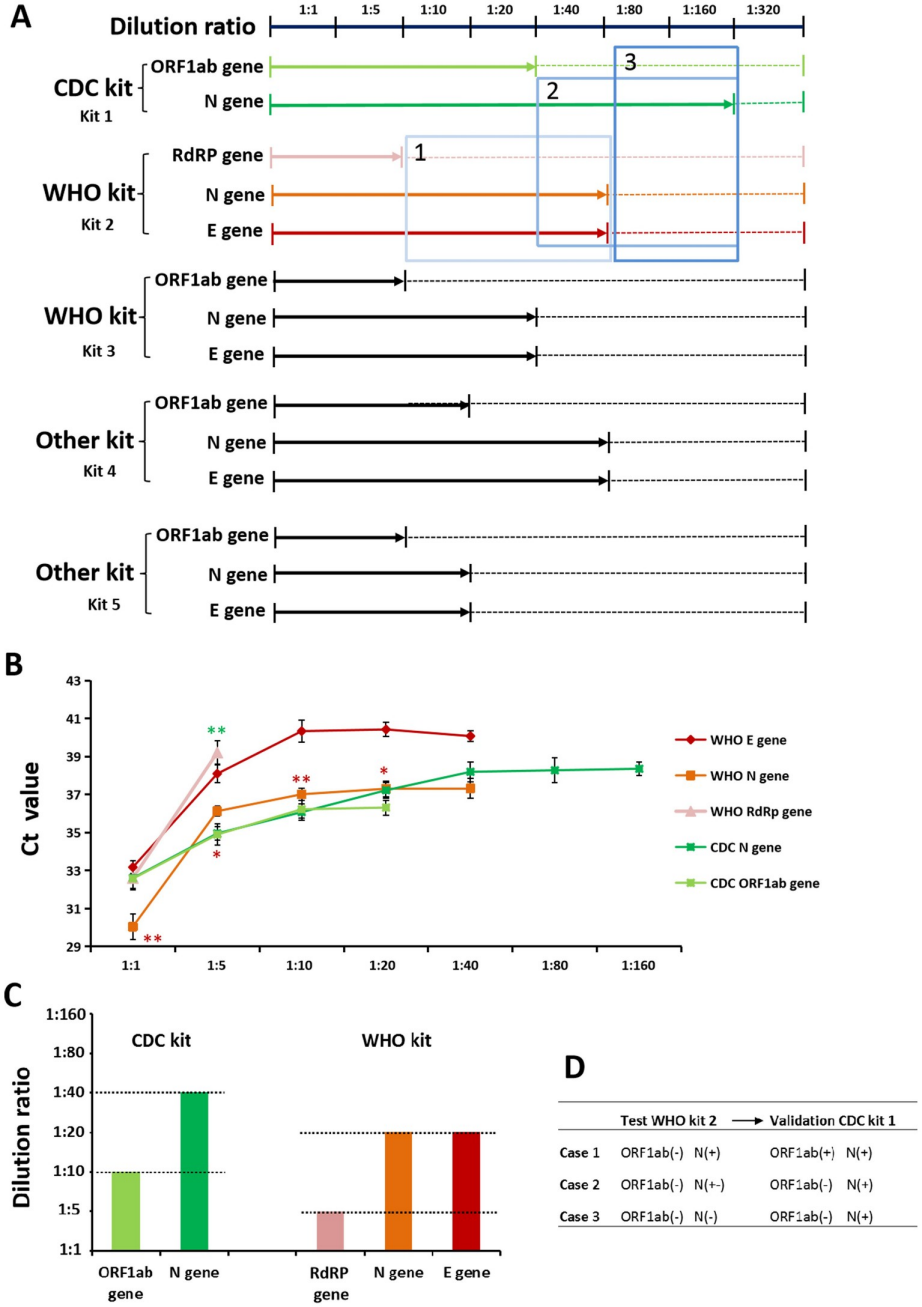
Sensitivity evaluation of SARS-CoV-2 detection kits. (A) Virus level that disappears with the increase in the dilution concentration in different kits. (B) The *Ct* value of the amplification curve was positively correlated with the dilution gradient. The red asterisk represents the comparison of *Ct* values of N gene, while the green asterisk represents the comparison of *Ct* values of ORF1ab/RdRp gene between the two kits. *P < 0.05; **P < 0.01. (C) Kit-1 has the highest sensitivity based on the verification of multiple positive samples. (D) Selection strategies of validation kit.

The lowest detection concentration is a critical performance parameter to evaluate the sensitivity of the kit. Viral RNA was extracted from a patient with SARS-CoV-2 and diluted according to the following proportion gradient: 1:5, 1:10, 1:20, 1:40, 1:80, 1:160, and 1:320. Each concentration was detected three times with five kits. Fig 1A shows a decreasing trend of virus level that disappears with the increase in the dilution concentration in different kits. The lowest detection concentration of *ORF1ab* and *N* genes was maximal in the kit-1, deeming it as the most sensitive to SARS-CoV-2, followed by kit-4, kit2, kit3, and kit5. In addition, the Ct value of the amplification curve was found to be positively correlated with the dilution gradient (Fig 1B). The comparison of the Ct values of each target gene in kit-1 and kit-2 revealed that the Ct value of *ORF1ab* and *N* genes in kit-1 were still within the positive reportable region at 1:20 and 1:160 dilution, respectively, while it exceeded the detection limit in kit-2 at 1:5 and 1:40 dilutions.

To further verify the applicability of the kits, another 9 positive samples were tested. The positive RNA extract was first quantified by digital PCR and then diluted to the same initial concentration. The results showed that the *ORF1ab* gene can still be reported as positive at 1:10 dilution and the *N* gene at 1:40 dilution (Fig 1C) with kit-1, while they exceeded the detection limit at 1:5 and 1:20, respectively, with kit-2. Hence, we presumed that kit-1 has the highest sensitivity based on the verification of multiple positive samples.

The above results showed that the sensitivity of *ORF1ab* gene was the lowest as compared to the other target genes of SARS-CoV-2, using all the detection kits. Then, if the *N* or *E* gene is positive and the *ORF1ab* gene is negative (three cases were presented in Fig 1A), how to judge the result and select the validation kit needs to be addressed. Our solution is as follows (Fig 1D): 1. Both *ORF1ab* and *N* genes can be converted to positive after verification with kit 1; 2. When *N* gene is in a suspicious region, as determined by kit 2, it can be converted to positive after verification with kit 1; 3. When the *N* gene is deemed negative with kit 2, it can be converted to positive after verification with kit 1.

#### Clinical validation and application

**Fig 2:**
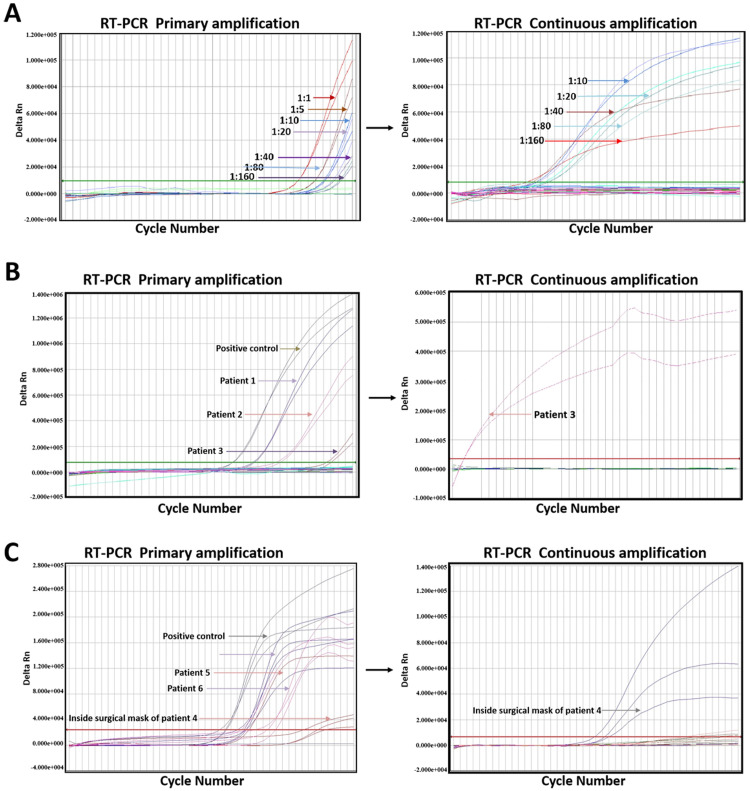
Strategies to reduce false negatives of SARS-CoV-2. (A) Continuous amplification of PCR products for gradient dilution samples. (B) Continuous amplification of PCR products for suspicious specimens. (C) Continuous amplification of PCR products for the environmental samples of 3 positive patients. Kit 1 was used for A and B, while kit 2 for C.

In addition to choosing a sensitive kit for validation, is there an easier method to increase the positive detection rate? First, the RT-PCR products of the above-diluted samples in the suspicious range were amplified for another 40 cycles. It was found that for the samples with dilution gradients of 1:10 and 1:20, the *ORF1ab* and *N* genes with large original amplified Ct values were expanded to the positive reporting region, while the other dilution gradients only with *N* or *E* genes were significantly amplified (Fig 2A). Moreover, 100 patients with clinical fever and dry cough, who were suspected to be infected with the SARS-CoV-2, were enrolled for RT-PCR, and 2 positive cases and 2 suspicious cases were identified (Table 2). Then, the suspicious cases were re-amplified and found to be positive by continuous amplification (Fig 2B and S1 Fig).

**Table 2:** Detection results of clinical environmental specimens by real-time PCR.

Specimens and values	Real-Time PCR						Continuous Real-Time PCR of +? results	
	Nasopharyngeal and oropharyngeal swabs (n = 14)		phone (n = 3)	doorknob (n = 3)	bed side (n = 3)	inner side of the mask (n = 3)	Nasopharyngeal and oropharyngeal swabs (n = 2)	inner side of the mask (n = 1)
**Positive test result, No. (%)**	12+ (85.8)	**2+? (14.2)**	0	0	0	**1+? (33)**	**2+ (100)**	**1+ (100)**
**Cycle threshold of target genes, mean (SD)**	30.1 (4.3)	36.5 (0.5)	>45	>45	>45	39 (1.0)	2.8 (0.4)	24 (1.0)
**Range of target genes**	25.8–35.0	36.0–37.0				38.0–40.0	2.4–3.2	23–25

Note: + positive; +? false negative.

Moreover, the environmental samples from 3 COVID-19 patients were subjected to nucleic acid testing. It was found that the sample inside the mask of 1 patient was weakly positive, which could be reported as positive after another re-amplification (Fig 2C). Further analysis revealed that each target gene could reach the amplification plateau by adding another 30 cycles. Also, we added the initial RT-PCR amplification products of positive patients into a new amplification reaction system, albeit the results were not reliable.

#### Strategies to reduce false negatives of SARS-CoV-2

Herein, we suggested that the sensitivity of the detection kits should be evaluated before their routine usage, and the sensitivity of the validation kit must be higher than that of the test kit. In mixed samples, the ultra-high sensitivity kit should be preferred. For specimens with suspicious interval region or single-channel positive results, the continuous amplification can be used to increase the detection rate of low viral load specimens and greatly reduce the false-negative rate of SARS-CoV-2.

## Discussion

As of September 2, 2020, statistical data showed that the global number of confirmed cases of COVID-19 had surpassed 25 million with >800,000 deaths [33]. With an increasing number of potential cases, the SARS-CoV-2 poses a major threat to global public health [34]. Currently, a large number of diagnostic tools, such as virus isolation, PCR-based assays, IHC, and antibody assays, have been developed across various diagnostic laboratories worldwide [35–39]. The detection rate of viral nucleic acid is closely related to the course of viral infection, and the optimal sampling time is uncertain; however, the period of the high viral load will be missed, resulting in false-negatives [40]. Although RT-PCR is challenged by the false-negative results [41], in view of the past major epidemic outbreaks [42], the method is still the preferred tool for detection. Therefore, how to ensure the accuracy of nucleic acid test results is currently under investigation.

Theoretically, the primers and probes of the target genes should have not only high specificity but also high sensitivity. The most conserved *ORF1ab* gene presents low sensitivity, while the other target genes, such as *N* or *E*, are less conservative but more sensitive. The current results showed that the lowest detection concentration of each kit differs substantially. If a low sensitivity kit is selected to detect SARS-CoV-2 infection, it may lead to false-negative results. In addition, multiple mutations were detected over the entire genomes of SARS-CoV-2 [43–45]. Variations are speculated to influence the rate of disease transmission and mortality [6, 30, 46] and also that the false-negative rate might be elevated if the mutation occurs at the current target genes of SARS-CoV-2. Except for the variations found throughout the genome, mutations of SARS-CoV-2 virus were also found at the primer- or probe-binding sites [47], which might lower the sensitivity of the current targeting genes. Furthermore, we speculated that the detection of Nsp1 could avoid false-negative results caused by mutations at the primer- or probe-binding sites [48]. Notably, the deletion of 382 nucleotides in the *ORF8* gene enhances the transcription of the downstream *N* gene [26], which might increase the false-negative detection rate of SARS-CoV-2. Thus, attention should be focused on the abnormally amplified *N* gene in clinical detection.

As a prospective method to resolve the dilemma of SARS-CoV-2 nucleic acid screening, mixed sample detection could be adopted for the low-risk population according to the regulations of the National Health Commission of the People’s Republic of China (http://www.nhc.gov.cn/yzygj/s7659/202008/fa5057afe4314ef8a9172edd6c65380e.shtml). The mixed samples include samples for mixed-collection and samples for mixed-detection. To simulate the mixed-collection, the swabs of confirmed positive and negative samples were mixed in a tube with a virus preservation solution. Subsequently, the *Ct* value of the amplification curve was unchanged, which indicated that the mixed-collection sample did not affect the positive detection rate. To simulate mixed-detection, the virus preservation solution of positive and negative samples was mixed equally and found that the *Ct* value increased correspondingly, which indicated that the sensitivity was reduced by mixed-detection (S1 Table). It is noteworthy that mixed-collection is not simply increase the number of swabs in the collection tube, but to carefully consider and design the specification and material of collection tube, the composition and content of virus preservation solution, the material of swab and the type of swab head, so as to ensure the accuracy of detection results. Therefore, in order to avoid false-negative results, sensitive detection kits should be used in mixed-detection.

Similar to all the viral nucleic acid testing projects, the RT-PCR results of SARS-CoV-2 are affected by various factors before, during, and after detection, and hence, laboratory quality-control measures should be implemented. In addition, extending amplification cycles do increase sensitivity, but may also reduce specificity. Nucleic acid samples of other respiratory pathogens with known concentrations were used for specificity testing and results showed there was no cross-reaction with other pathogens and the specificity did not decrease (S2 Table). Moreover, the continuous amplification and other detection methods of SARS-CoV exhibited false-positive results [49, 50]; thus, we recommended using it only when the amplification curve of the target gene is in the specious region.

## Conclusions

The emergence of mixed sample detection challenges the sensitivity of nucleic acid detection kits. We speculated that the detection of mixed samples is only applicable to low-risk populations using the high-sensitivity kits. Importantly, the antibody and nucleic acid tests should complement each other to improve the diagnosis, especially to screen the asymptomatic patients better and reduce the false-negative phenomenon of “false recovered patients” or premorbid patients with low virus latency.

## Supporting information

S1 Fig Continuous amplification of PCR product for another suspected specimen. (TIF)

S1 Table The results of samples for mixed-collection and mixed-detection with kit-1. (PDF)

S2 Table Cross-reactive results with other pathogens. (PDF)
