# Supplementary figures and images for: Sensitivity evaluation of 2019 novel coronavirus (SARS-CoV-2) RT-PCR detection kits and strategy to reduce false negative
Source: PLoS One. 2020 Nov 18;15(11):e0241469. doi: 10.1371/journal.pone.0241469 (PMC7673793; doi:10.1371/journal.pone.0241469)

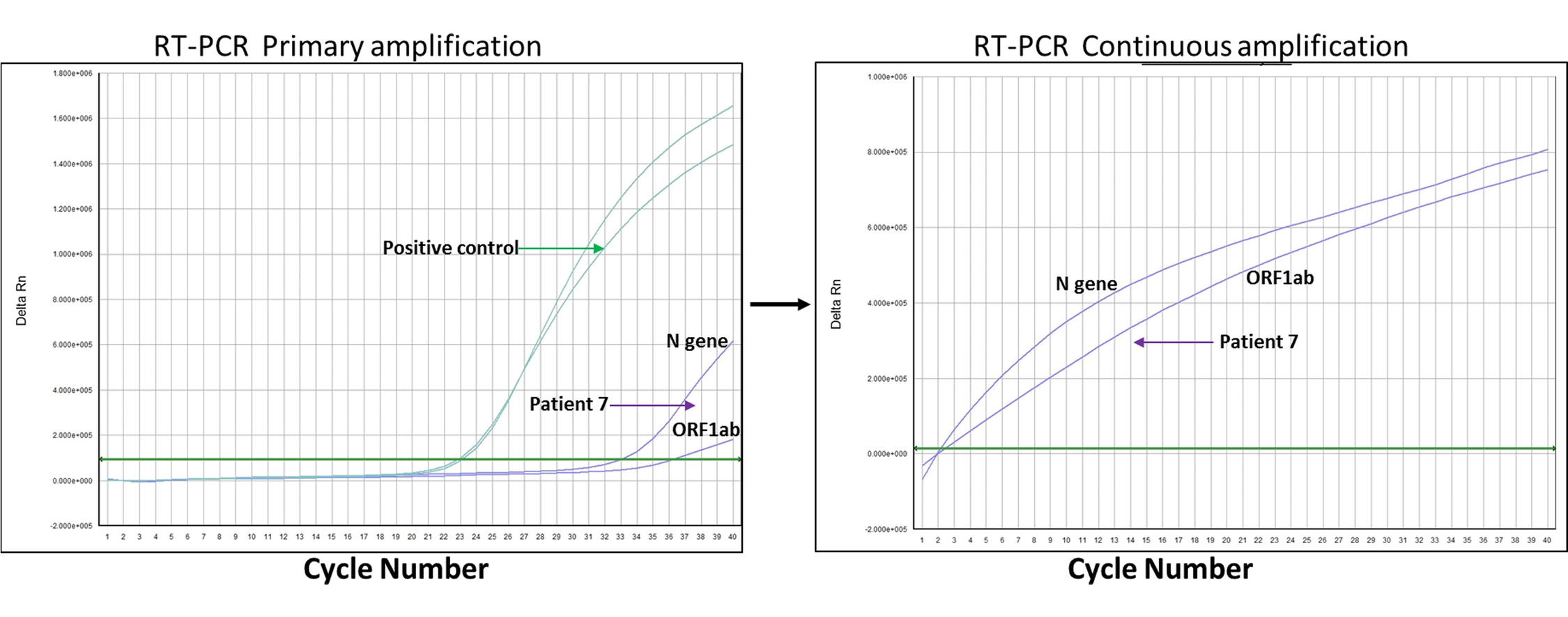

Supplement: S1 Fig — (TIF) [file pone.0241469.s001.tif]
