# Supplementary material for: Sensitivity evaluation of 2019 novel coronavirus (SARS-CoV-2) RT-PCR detection kits and strategy to reduce false negative
Source: PLoS One. 2020 Nov 18;15(11):e0241469. doi: 10.1371/journal.pone.0241469 (PMC7673793; doi:10.1371/journal.pone.0241469)
Supplement: S1 Table — (PDF) [file pone.0241469.s002.pdf]

Supplemental table 1

The results of samples for mixed-collection and mixed-detection with kit-1.

Specimens	Results criteria	Single detection result	Detection result of mixed-collection (1:10)	Detection result of mixed-detection (1:10)
**1**	**(+/-)**	+	+	+
	**Ct value**	32	32	35
**2**	**(+/-)**	+	+	-
	**Ct value**	34	34	37
**3**	**(+/-)**	+	+	-
	**Ct value**	33	33	37

Mixed-collection: 10 swabs of 1 positive and 9 negative samples mixed in a virus preservation solution tube.
Mixed-detection: equally mixture of virus preservation solution with 1 positive and 9 negative samples.
