# Supplementary material for: Sensitivity evaluation of 2019 novel coronavirus (SARS-CoV-2) RT-PCR detection kits and strategy to reduce false negative
Source: PLoS One. 2020 Nov 18;15(11):e0241469. doi: 10.1371/journal.pone.0241469 (PMC7673793; doi:10.1371/journal.pone.0241469)
Supplement: S2 Table — (PDF) [file pone.0241469.s003.pdf]

Supplemental table 2

Cross-reactive results with other pathogens.

Samples	Nucleic acid concentration	Repeat 1	Repeat 2	Repeat 3
**Negative sample**	-	-	-	-
**Negative control**	-	-	-	-
**Positive control**	2×10^5	+	+	+
**HCoV-229E**	1.38×10^8	-	-	-
**HCoV-NL63**	7.96×10^7	-	-	-
**HCoV-OC43**	2.11×10^8	-	-	-
**HCoV-HKU1**	3.95×10^8	-	-	-
**SARS-CoV**	8.33×10^8	-	-	-
**MERS-CoV**	8.65×10^7	-	-	-
**Novel influenza virus A/H1N1/ H1**	7.61×10^7	-	-	-
**Seasonal influenza virus H1N1**	3.36×10^9	-	-	-
**H3N2**	3.76×10^8	-	-	-
**H5N1**	5.97×10^8	-	-	-
**H7N9**	6.51×10^9	-	-	-
**Influenza B Yamagata**	7.78×10^7	-	-	-
**Influenza B Victoria**	9.03×10^7	-	-	-
**Respiratory syncytial virus A**	4.76×10^7	-	-	-
**Respiratory syncytial virus B**	6.18×10^6	-	-	-
**parainfluenza virus virus 1**	4×10^7	-	-	-
**parainfluenza virus 2**	1.66×10^7	-	-	-
**parainfluenza virus 3**	6.87×10^7	-	-	-
**Rotavirus**	2.98×10^8	-	-	-
**Norovirus**	2.73×10^7	-	-	-
**Mumps virus**	9.53×10^7	-	-	-
**Varicella-zoster virus**	9.65×10^6	-	-	-
**Rhinovirus type A**	2×10^7	-	-	-
**Rhinovirus type B**	5.31×10^7	-	-	-
**Rhinovirus type C**	6.53×10^8	-	-	-
**Adenovirus type 1**	7.35×10^7	-	-	-
**Adenovirus type 2**	3.36×10^7	-	-	-
**Adenovirus type 3**	7.32×10^6	-	-	-
**Adenovirus type 4**	7.97×10^7	-	-	-
**Adenovirus type 5**	1.38×10^9	-	-	-
**Adenovirus type 7**	7.96×10^7	-	-	-
**Adenovirus type 55**	1.34×10^7	-	-	-
**Enterovirus type A**	1.22×10^9	-	-	-
**Enterovirus type B**	2.36×10^8	-	-	-
**Enterovirus type C**	2.85×10^7	-	-	-
**Enterovirus type D**	3.27×10^8	-	-	-
**EB virus**	8.31×10^8	-	-	-
**Measles virus**	6.2×10^7	-	-	-
**HCMV**	1.34×10^7	-	-	-
**Aspergillus fumigatus**	7.07×10^9	-	-	-
**Candida albicans**	1.34×10^8	-	-	-
**Candida glabrata**	1.22×10^9	-	-	-
**Mycoplasma pneumoniae**	5.74×10^7	-	-	-
**Chlamydia pneumoniae**	2.7×10^7	-	-	-
**legionella**	7.71×10^7	-	-	-
**Bordetella pertussis**	3×10^9	-	-	-
**Haemophilus influenzae**	2.73×10^8	-	-	-
**Staphylococcus aureus**	9×10^7	-	-	-
**Streptococcus pneumoniae**	7.39×10^9	-	-	-
**Streptococcus pyogenes**	5.96×10^7	-	-	-
**Klebsiella pneumoniae**	6.86×10^8	-	-	-
**Mycobacterium tuberculosis**	1.91×10^8	-	-	-
**Cryptococcus Neoformans**	7.97×10^7	-	-	-
